# The Effectiveness of Hypomethylating Agents in Elderly Patients With Acute Myeloid Leukemia: Insights From a Single-Center Experience

**DOI:** 10.7759/cureus.82957

**Published:** 2025-04-24

**Authors:** Cristina Negotei, Iuliana Mitu, Oana Stanca, Silvana Angelescu, Mihai-Emilian Lapadat, Cristian Barta, Nicoleta M Berbec, Andrei Colita

**Affiliations:** 1 Department of Hematology, Carol Davila University of Medicine and Pharmacy, Bucuresti, ROU; 2 Clinic of Hematology, Coltea Clinical Hospital, Bucharest, ROU; 3 Department of Hematology, Carol Davila University of Medicine and Pharmacy, Bucharest, ROU

**Keywords:** acute myeloid leukemia, elderly patients, hypomethylating agents, overall survival, venetoclax

## Abstract

Introduction: Acute myeloid leukemia (AML) is an aggressive hematologic malignancy mainly affecting older adults. It presents significant challenges due to unfavorable cytogenetic characteristics and associated comorbidities, which restrict treatment options. Addressing these complexities is essential for improving patient outcomes. Hypomethylating agents (HMAs) such as azacitidine (AZA) and decitabine (DAC) are standard treatments for elderly patients unfit for intensive chemotherapy. The introduction of Venetoclax (Ven), a selective BCL-2 inhibitor, has shown promise in improving treatment outcomes.

Methods: A retrospective analysis was conducted on 82 elderly AML patients treated at the Coltea Clinical Hospital, Bucharest, Romania, between January 2017 and December 2023. Patients received non-intensive chemotherapy with either AZA, DAC, LDAC, or their combination with Venetoclax. Clinical, cytogenetic, and molecular parameters were documented, and survival outcomes were analyzed using the Kaplan-Meier method. Statistical significance was assessed using the log-rank test and Cox proportional hazard regression.

Results: The median overall survival (OS) was seven months among the patients receiving a single line of therapy. By contrast, in those treated with a second-line regimen that included Venetoclax, OS increased to 11 months. Although the sample size was small, this difference reached statistical significance (p = 0.038). Transfusion independence was significantly higher in Venetoclax-treated patients.

Conclusion: Hypomethylating agents, in combination with Venetoclax, have emerged as the standard of care for elderly patients with AML, offering superior survival and response rates. Nevertheless, further therapeutic advancements are crucial to enhance treatment outcomes in this population.

## Introduction

Acute myeloid leukemia (AML) is an aggressive blood cancer caused by the clonal expansion of immature myeloid cells, which impairs normal hematopoiesis and leads to bone marrow failure. It predominantly affects older adults and is driven by genetic mutations that promote uncontrolled cell growth [1,2].

Treating elderly patients with AML requires a personalized approach based on age, performance status, and comorbidities to determine treatment suitability. Elderly patients (≥60 years) have a much poorer outlook than younger patients. They have a three-year survival rate of only 9-10%, whereas younger patients have nearly a 50% survival rate [3]. This comparison refers to survival rates observed in patients treated with intensive chemotherapy, which is often not feasible in elderly individuals due to comorbidities and frailty. This study solely included elderly patients (aged 65 and older) who were not eligible for intensive chemotherapy or stem cell transplantation. Venetoclax was given exclusively as part of low-intensity regimens, following current clinical guidelines for unfit AML patients. In our country, the combination of Venetoclax with hypomethylating agents has been officially approved since 2022, specifically for patients not suitable for intensive chemotherapy, which reinforces its indication in this context.

Traditional low-intensity treatments, such as low-dose cytarabine or hypomethylating agents (HMAs), lower toxicity but often achieve less than 30% remission rates. The median overall survival for these treatments is less than one year [4]. Older AML patients frequently present with adverse cytogenetics, secondary AML, and comorbidities, which limit their ability to tolerate intensive chemotherapy. As a result, treatment has shifted toward targeted therapies; however, the prognosis remains poor, with nearly 70% of patients over 65 dying within the first year of diagnosis [5,6,7]. Low-dose cytarabine (LDAC) was considered a standard treatment for patients classified "unfit" for intensive chemotherapy. Clinical evidence shows that LDAC provides notable advantages over best supportive care and hydroxyurea for those who cannot tolerate intensive therapy [8]. Changes in gene regulation, especially unusual DNA methylation, significantly impact tumor suppressor genes and oncogenes in AML. These changes can be reversed, making them potential targets for treatment. Azacitidine (AZA) and decitabine (DAC) are pyrimidine nucleoside analogs that inhibit DNA methyltransferase and interfere with DNA methylation. Their effectiveness, manageable side effects, and suitability for outpatient treatment make them appropriate for older patients with AML [6].

AZA and DAC exhibit differences in their structure and mechanisms of action. AZA mainly integrates into RNA, thereby disrupting ribosome assembly and the process of protein synthesis. A smaller proportion of AZA binds to DNA, which contributes to the reactivation of tumor suppressor genes [9]. Decitabine is an inhibitor of DNA methyltransferases (DNMTs), resulting in DNA hypomethylation, particularly in the promoter regions of genes. This process may enable the re-expression of previously silenced tumor suppressor genes. In addition, decitabine induces cytotoxicity by integrating into DNA and forming DNA-DNMT adducts that disrupt DNA synthesis during cell replication, ultimately leading to apoptosis [10].

Although widely used, no randomized trials have directly compared their efficacy, and the choice of agents remains dependent on the physician's judgment. Hypomethylating agents are now standard therapy for older AML patients who are ineligible for intensive chemotherapy (IC) [9]. Eligibility for this treatment is determined using various patient evaluation systems, with the most commonly used being the Eastern Cooperative Oncology Group (ECOG) Performance Status, the Charlson Comorbidity Index (CCI), and the Hematopoietic Cell Transplantation Comorbidity Index (HCT-CI). The ECOG Performance Status is a standardized scale used to assess a patient’s functional capacity and how their illness affects daily activities, with lower scores linked to reduced survival and response rates [11]. CCI quantifies the impact of pre-existing conditions, such as heart disease and diabetes, to predict long-term survival and guide treatment decisions [12]. HCT-CI helps estimate early mortality risk in older leukemia patients, emphasizing the importance of individualized medical management [13]. The integration of Venetoclax (Ven) into low-intensity treatment options presents an opportunity to improve patient outcomes in AML. Venetoclax is a highly selective inhibitor of BCL-2 that plays a role in the modulation of apoptosis by changing mitochondrial membrane permeability. The US Food and Drug Administration approved these drug combinations for patients over 75 years old or for those who cannot tolerate IC [14], [15]. In 2018, Glasdegib, a Hedgehog pathway inhibitor that blocks the SMO (Smoothened) protein, was approved in combination with LDAC in patients with newly diagnosed AML aged ≥75 years with multiple comorbidities. The BRIGHT study AML 1003 focused on elderly patients and those with preexisting health conditions who have AML. The trial included 115 patients aged 75 and older or 55 and older with existing health issues. Participants received either Glasdegib with LDAC or LDAC alone. The results showed that the combination therapy improved overall survival (OS) significantly [16].

The aim of this study was to assess the Coltea Hematology Department's experience with hypomethylating agents in elderly patients diagnosed with AML and to compare the results with existing literature.

## Materials and methods

We conducted a retrospective survey to evaluate the clinical outcomes of elderly, medically unfit patients who were diagnosed with AML, including both de novo and secondary cases. Patients received non-intensive chemotherapy at Coltea Clinical Hospital’s Hematology Department in Bucharest, Romania. This research aimed to evaluate the general prognosis for these patients based on clinical and demographic factors.

All patients diagnosed with AML aged 65 years or older who underwent non-intensive chemotherapy were included. No young adult patients were enrolled. Considering the specifics of the research, patients were treated with azacitidine, decitabine, venetoclax, low-dose cytarabine, and glasdegib. Treatment choices followed institutional protocols for patients unfit for intensive chemotherapy. Venetoclax was added to hypomethylating agents in selected cases based on clinical condition and guideline-based recommendations.

We documented their age, gender, date of arrival at our clinic, ECOG Performance Status, HCT-CI, CCI, comorbidities, left ventricular ejection fraction (LVEF), type of AML, hemoglobin level, platelet count, leukocytes, blasts in bone marrow, molecular biology, karyotype, treatment and response to treatment, disease and treatment complications, cause of death, and date of death. Molecular and cytogenetic evaluation at our institution included conventional karyotyping, PCR-based mutation testing, and fluorescence in situ hybridization (FISH) in selected cases. The analysis process involved reviewing patients’ medical records and entering the data into an MS Excel spreadsheet (Microsoft Corp., USA) for further examination.

In our clinic, 82 patients have undergone frontline therapy. The distribution of treatments among these patients is as follows: 24 patients received azacitidine, 32 patients received decitabine, 15 patients were treated with hypomethylating agents combined with venetoclax, and 11 patients were given LDAC. Treatment response was assessed according to standard hematologic criteria, including complete remission (CR: <5% blasts in bone marrow and recovery of peripheral counts), complete remission with incomplete hematologic recovery (CRi), and partial response (PR). Patients not meeting these criteria were considered non-responders. These definitions were applied uniformly across all treatment arms. Of the 24 patients receiving azacitidine, 12 achieved a response, while 12 did not. Among those who responded, six relapsed, and six did not. Of the 32 patients receiving decitabine, 15 experienced a therapeutic response, and 17 did not. Among the responders, five relapsed, while 10 did not. Out of the 15 patients receiving hypomethylating agents in combination with venetoclax, eight showed a response, and seven did not. Of those who responded, one experienced a disease relapse, while seven did not relapse.

All 11 patients undergoing therapy with LDAC, either in monotherapy or in combination with glasdegib, failed to show a therapeutic response and, unfortunately, died. Following this analysis, 47 patients had no therapeutic response, while 12 out of the 35 patients who achieved therapeutic response experienced a relapse. Among the relapsed patients, 12 proceeded to receive second-line therapy, along with an additional four patients from the group of 47 who were refractory. In summary, all 82 patients received frontline therapy, 66 undergoing a single therapeutic line, while 16 received two.

Each therapeutic option was evaluated based on important factors such as age, ECOG performance status, CCI score, and HCT-CI. The statistical analyses were conducted using SPSS version 26. We used the Kaplan-Meier analysis to determine the average overall survival time and the average survival time without disease progression (PFS) for the entire study group without any adjustments. The median value was used to estimate the average survival time. Although we used an unadjusted Kaplan-Meier analysis for estimating survival outcomes in this study, future research will aim to include multivariate models to adjust for potential confounding variables. For a comparative analysis of survival time, we used the Kaplan-Meier comparative analysis with the log-rank (Mantel-Cox) test based on demographic risk factors and other clinical characteristics. To assess the impact of a risk factor on survival, we calculated the hazard ratio (HR) using Cox proportional hazard regression analysis. We considered a p-value of less than 0.05 to be statistically significant.

## Results

Between January 2017 and December 2023, our cancer center provided non-intensive treatment to 87 patients diagnosed with AML.

The study involved a cohort of 87 patients aged 65 and older who were diagnosed with AML. Unfortunately, five patients were lost to follow-up, leading to a statistical analysis based on a total of 82 patients. The diagnostic process included a bone marrow cytological examination, bone marrow flow cytometry, molecular biology, and karyotyping. In some cases, the prognostic classification was based on cytogenetic or molecular alterations according to the 2017 European LeukemiaNet (ELN) classification [17]. For prognostic stratification, both cytogenetic and molecular biology analyses were considered. If one analysis could not be performed due to limited resources, stratification was based on the available analysis instead.

The research cohort for this study included a total of 82 patients who presented with the following characteristics: 45 patients were aged between 65 and 74 years, while 37 patients were older than 75 years (p-value 0.377). The gender distribution consisted of 52 males and 30 females, with a p-value of 0.015. The study found that 41.5% of patients had de novo leukemia, while the rest, 58.5%, had secondary leukemia after myelodysplasia or treatment-related (p < 0.122). Patients were stratified by risk group based on cytogenetic and molecular biology examinations, where applicable. A total of 49 individuals (59.8%) had a molecular biology examination, and 41 individuals (50.0%) had a cytogenetic examination.

Of the 41 patients evaluated, 15 had a normal karyotype, 18 had complex karyotypes, and eight had other cytogenetic abnormalities, including trisomy 3, trisomy 11, add(15), del(6), del(19), del(20), add(22), del(5q), and t(8;21) translocation. The patients were classified into risk categories as follows: 51.2% were in the intermediate-risk category, 46.4% in the adverse-risk category, and only one patient was classified in the favorable risk category (p < 0.001).

All patients had comorbidities, with 66 out of 82 individuals having more than three (p < 0.001); 58.5% of the patients had normal LVEF. A significantly higher ratio of patients had leukocytes <10,000 (62/82; p < 0.001), platelets < 100,000 (66/82; p < 0.002), and a percentage of blasts <50% (55/82; p < 0.002). More individuals had a CCI greater than 4 (78 out of 82; p < 0.001). In the HCT-CI analysis, the intervals 1-4 and >4 weights were approximately equal (40 vs. 42; p -0.825). The highest proportion of scores was greater than 4, with 42 out of 82 participants (51.3%), while the lowest proportion was for scores in the range of 1 to 2, with 12 out of 82 participants (14.6%) (p < 0.001).

The ECOG scores were equally distributed among patients, with 41 cases (50%) scoring between 0 and 1 and 41 cases (50%) scoring between 2 and 4, as shown in Table 1.

**Table 1:** Characteristics of patients diagnosed with AML who had undergone non-intensive chemotherapy treatment Abbreviations: AML, acute myeloid leukemia; ECOG, Eastern Cooperative Oncology Group; CCI, Charlson Comorbidity Index; HCT-CI, Hematopoietic Cell Transplantation Comorbidity Index; LVEF, left ventricular ejection fraction; chi-square test: χ2—test value, df—degree of freedom, p-value.

Variable		Number of patients	Percentage (%)	Chi-square test of proportion		
				X^2^	df	p
Age (years)	65-74	45	54.90	0.78	1	0.377
	≥75	37	45.10			
Gender	Male	52	63.40	5.90	1	0.015
	Female	30	36.6			
AML classification	De novo	34	41.5	2.39	1	0.122
	Secondary	48	58.5			
Karyotype	Normal	15	36.60	3.85	2	0.146
	Other cytogenetic abnormalities	8	19.50			
	Complex	18	43.90			
Risk stratification	Favorable	1	2.40	17.76	2	0.001
	Intermediate	21	51.20			
	Adverse	19	46.40			
Molecular Biology	Yes	49	59.80	3.12	1	0.077
	No	33	40.20			
The presence of mutations	Yes	5	10.20	31.04	1	<0.001
	No	44	89.80			
Risk stratification	Favorable	3	6.20	75.59	2	<0.001
	Intermediate	45	91.80			
	Adverse	1	2.00			
ECOG	0-1	41	50.00	0	1	-
	2-4	41	50.00			
CCI	4	4	4.90	66.78	1	<0.001
	>4	78	95.10			
HCT-CI	1-2	12	14.60	16.49	2	<0.001

Among 82 patients, 11 were treated with LDAC, while the remaining 71 received hypomethylating agents, either in monotherapy or in combination with venetoclax. The cohort included 52 men and 30 women in terms of gender distribution, with a p-value of 0.243, as Table 2 displays.

**Table 2:** Treatment distribution for patients receiving frontline therapy Abbreviations: AML, acute myeloid leukemia; Aza, azacytidine, Dac, decitabine, Ven, venetoclax; LDAC, low-dose cytarabine, glasdegib, MDS, myelodysplastic syndrome; chi-square test: χ2—test value, df—degree of freedom, p-value.

Factor		Aza	Dac	Aza/Dac + Ven	LDAC/ LDAC±Glasdegib	Chi-square test		
		N (%)	N (%)	N (%)	N (%)	χ^2^	df	p
Gender	Male	17 (70.8)	17 (53.1)	12 (80.0)	6 (54.5)	4.18	3	0.243
	Female	7 (29.2)	15 (46.9)	3 (20.0)	5 (45.5)			
Age	65 – 74	15 (62.5)	20 (62.5)	8 (53.3)	2 (18.2)	7.31	3	0.063
	≥ 75	9 (37.5)	12 (37.5)	7 (46.7)	9 (81.8)			
Post-MDS	Yes	15 (62.5)	7 (21.9)	5 (33.3)	4 (36.4)	9.82	3	0.020
	No	9 (37.5)	25 (78.1)	10 (66.7)	7 (63.6)			
AML classification	De novo	3 (12.5)	18 (56.3)	7 (46.7)	6 (54.5)	12.12	3	0.007
	Secondary	21 (87.5)	14 (43.7)	8 (53.3)	5 (45.5)			
Karyotype	Normal	3 (18.8)	4 (33.3)	8 (72.7)	0 (0.0)	13.89	6	0.031
	Other cytogenetic abnormalities	7 (43.8)	7 (58.3)	3 (27.3)	1 (50.0)			
	Complex	6 (37.4)	1 (8.4)	0 (0.0)	1 (50.0)			
Cytogenetic risk prognosis	Favorable	0 (0.0)	0 (0.0)	0 (0.0)	0 (0.0)	4.58	3	0.205
	Intermediate	8 (53.3)	5 (41.7)	8 (72.7)	0 (0.0)			
	Adverse	7 (46.7)	7 (58.3)	3 (27.3)	2 (100.0)			
Molecular biology risk prognosis	Favorable	2 (16.7)	1 (4.5)	0 (0.0)	0 (0.0)	4.62	6	0.593
	Intermediate	10 (83.3)	20 (91.0)	11 (100.0)	4 (100.0)			
	Adverse	0 (0.0)	1 (4.5)	0 (0.0)	0 (0.0)			

Cytogenetic analysis (karyotyping) was performed for only 41 patients, among whom 18 (43.9%) were identified as having a complex karyotype. Stratification by prognosis group showed that the majority of patients were classified in the intermediate-adverse risk category, with a p-value of 0.001. Molecular biology testing was performed on 49 patients, and of these, 45 were classified as intermediate risk. Only five patients had identified mutations, which included FLT3-ITD, RUNX1-RUNX1T1, and JAK2.

Several key points are important to highlight in the study involving 11 patients who received LDAC therapy. Of these patients, nine were older than 75 years, and 10 had an ECOG performance status between 2 and 4. In addition, all patients had a CCI score greater than 4 and an HCT-CI score above 3. Furthermore, two patients were categorized in the adverse prognosis group. As a result, all patients were classified as non-responders, and nine of them died within the first 60 days of treatment.

Treatment responses in patients who received frontline therapy are presented as follows: Decitabine was the most commonly used hypomethylating agent, with 46.9% of patients achieving either a complete remission, complete remission with incomplete hematologic recovery, or partial response, while 53.1% of patients showed no response. In the case of azacitidine, the response rate was equal among patients who responded to treatment and those who did not (50% vs. 50%). The combination of VEN with an HMA resulted in a response rate of 53.3%, as presented in Table 3.

**Table 3:** An analysis of treatment responses in patients receiving frontline therapy with HMAs, either alone or in combination with Venetoclax. Abbreviations: Aza, azacitidine; Dac, decitabine; Ven, venetoclax; LDAC, low-dose cytarabine, CR, complete remission; CRi, complete remission with incomplete count recovery; PR, partial remission; PFS, progression free survival; chi-square test: χ2—test value, df—degree of freedom, p-value.

Factor		Aza	Dac	Aza/Dac + Ven	Chi-square test		
		N (%)	N (%)	N (%)	χ^2^	df	p
Type of answer	Therapeuticresponse (CR + CRi + PR)	12 (50.0)	15 (46.9)	8 (53.3)	0.18	2	0.915
	No response	12 (50.0)	17 (53.1)	7 (46.7)			
Relapse	Yes	6 (50.0)	5 (33.3)	1 (12.5)	2.28	2	0.320
PFS	Min	8	2	1			
	Max	28	36	16			
	Average	12.25	9.33	7.75			
	Std. deviation	9.95	8.71	4.83			
	Median	7.00	8.00	8.00			
Survivaltime	< 6 months	10 (41.7)	18 (56.3)	5 (33.3)	2.49	2	0.287
	≥ 6 months	14 (58.3)	14 (43.7)	10 (66.7)			

A statistically significant lower proportion of patients achieved red blood cell transfusion independence (RBC-TI) (8/56 - 14.28%). However, a significantly higher proportion of RBC-TI was observed in patients receiving venetoclax (8/15 - 53.3%) compared to those treated with azacitidine (2/24 - 8.33%) or decitabine (6/32 - 18.75%) in monotherapy (p = 0.011).

Patients aged 65 to 74 years have an odds ratio (OR) of 3.38 (B = 1.22; SE = 0.48; Wald = 6.52; df = 1; p = 0.011; Exp(B) = 3.38), indicating that they are 3.38 times more likely to achieve a response compared to those aged over 75 years. Patients with ECOG of 0 to 1 have an odds ratio (OR) of 8.00 (B = 2.07; SE = 0.51; Wald = 16.31; df = 1; p < 0.001; Exp(B) = 8.00), so they are eight times more likely to achieve a response than those with ECOG scores of 2 to 4. In addition, these patients have an OR of 4.64 (B = 1.54; SE = 0.48; Wald = 10.45; df = 1; p = 0.001; Exp(B) = 4.64), indicating that they are 4.64 times more likely to survive for more than six months compared to those with ECOG scores of 2 to 4. Patients with normal LVEF have an OR of 20.67 (B = 3.03; SE = 0.68; Wald = 19.97; df = 1; p < 0.001; Exp(B) = 20.68), which means that they are 20.67 times more likely to achieve a response than those with low LVEF. Furthermore, they have an OR of 7.00 (B = 1.97; SE = 0.51; Wald = 14.86; df = 1; p = 0.001; Exp(B) = 7.15), indicating that they are seven times more likely to survive for more than six months compared to patients with low LVEF.

**Figure 1:**
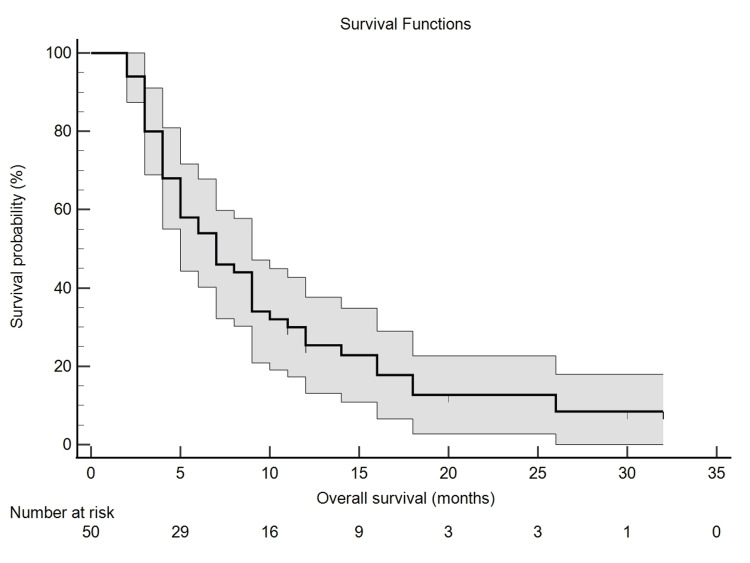
Median overall survival of seven months following first-line therapy, based on Kaplan-Meier analysis with 95% confidence intervals.

The study indicated that the overall survival (OS) duration ranged from 0 to 32 months after a single line of treatment. The Kaplan-Meier curve indicated a median OS of seven months (7.00 ± 1.51) with a 95% CI ranging from four to nine months (95% CI: 4.04-9.96), as Figure 1 exhibits.

Relapsed/refractory patients who received second-line therapy, consisting either of LDAC alone or of LDAC/HMAs in combination with venetoclax were compared. Patients with acute myeloid leukemia secondary to MDS were more likely to receive combination therapy that included venetoclax (87.5%). The association between post-MDS status and treatment choice was statistically significant (p = 0.003). Moreover, the preference for venetoclax-based regimens in de novo AML was statistically significant (p = 0.002), likely reflecting higher response rates in this subgroup. No statistically significant differences were observed regarding karyotype and cytogenetic risk prognosis (p = 0.167), as detailed in Table 4.

**Table 4:** Clinical characteristics of refractory/relapsed patients receiving a second-line of treatment Abbreviations: AML, acute myeloid leukemia; MDS, myelodysplastic syndrome; LDAC, low-dose cytarabine; HMAs, hypomethylating agents; Aza, azacitidine; Dac, decitabine; Ven, venetoclax; chi-square test: χ2—test value, df—degree of freedom, p-value.

Variable		LDAC	HMAs/LDAC+Ven	Chi-square test of proportion		
		n/N	n/N	c^2^	df	p
Age (years)	65-74	7/8	8/8	1.07	1	0.302
	≥75	1/8	0/8			
Gender	Male	4/8	6/8	1.07	1	0.302
	Female	4/8	2/8			
Post-MDS	Yes	1/8	7/8	9.00	1	0.003
	No	7/8	1/8			
AML classification	De novo	0/8	6/8	9.60	1	0.002
	Secondary	8/8	2/8			
Karyotype	Normal	1/6	4/5	5.08	2	0.079
	Complex	3/6	0/5			
	Other cytogenetic abnormalities	2/6	1/5			
Cytogenetic risk prognosis	Favorable	0/6	1/5	2.86	1	0.167
	Intermediate	3/6	4/5			
	Adverse	3/6	0/5			
Molecular biology risk prognosis	Favorable	0/5	1/7	0.78	1	0.583
	Intermediate	5/5	6/7			
	Adverse	0/5	0/7			

A statistically significant relationship between the types of treatment and the corresponding responses, with a p-value of 0.055. All patients in the LDAC monotherapy arm did not respond; all died, with a survival rate of less than six months. 3 out of 8 patients (37.5%) in the HMAs/LDAC + Ven group achieved a therapeutic response (CR + CRi + PR). No significant differences were found between the groups in relapse rates or overall mortality, with all p-values exceeding 0.05. These findings are detailed in Table 5, indicating that adding Venetoclax to HMAs/LDAC therapy may provide a clinical benefit for this patient population.

**Table 5:** Treatment and response in R/R patients Abbreviations: LDAC, low-dose cytarabine; HMAs, hypomethylating agents, Ven, Venetoclax; CR, complete remission; CRi, complete remission with incomplete count recovery; PR, partial remission; chi-square test: χ2—test value, df—degree of freedom, p-value.

Factor		LDAC	HMAs/LDAC+Ven	Chi-square test		
		n/N	n/N	χ2	df	p
Type of answer	Therapeutic response (CR + CRi + PR)	0/8	3/8	3.69	1	0.055
	No response	8/8	5/8			
Relapse	yes	--	2/3	2.29	1	0.233
Survival time	< 6 months	7/8	6/8	0.41	1	0.500
	≥ 6 months	1/8	2/8			
Death	yes	8/8	7/8	1.07	1	0.500

Patients who benefited from two lines of treatment (15 out of 16, aged between 65 and 74 years) were significantly younger (p < 0.001) compared to those who only received one line of therapy (36 out of 66, aged over 75 years). The ECOG performance status of patients benefiting from second-line therapy (12 out of 16, with scores of 0 or 1) was significantly better (p = 0.024) than that of patients who only received one line of therapy (37 out of 66, with scores ranging from 2 to 4). The proportion of individuals with an abnormal left ventricular ejection fraction (LVEF) among those benefiting from second-line therapy (3 out of 16, or 18.7%) was significantly lower (p = 0.035) than for those who only received on line of treatment (31 out of 66, or 47.0%), as illustrated in Table 6.

**Table 6:** Characteristics of patients based on the number of therapeutic lines Abbreviations: AML, acute myeloid leukemia; MDS, myelodysplastic syndrome; ECOG, Eastern Cooperative Oncology Group; CCI, Charlson Comorbidity Index; HCT-CI, Hematopoietic Cell Transplantation Comorbidity Index; LVEF, left ventricular ejection fraction; chi-square test: χ2—test value, df—degree of freedom, p-value.

Variable		One line		Two lines		Chi-square test of proportion		
		n	%	n	%	c^2^	df	p
Age (years)	65-74	30	45.5	15	93.7	12.13	1	<0.001
	≥75	36	54.5	1	6.3			
Gender	Male	42	63.6	10	62.5	0.01	1	0.575
	Female	24	36.4	6	37.5			
Post-MDS	Yes	23	34.8	8	50.0	1.26	1	0.201
	No	43	65.2	8	50.0			
AML classification	De novo	28	42.4	6	37.5	0.13	1	0.474
	Secondary	38	57.6	10	62.5			
Cytogenetic test	Yes	30	45.5	11	68.8	2.8	1	0.081
	No	36	54.5	5	31.2			
Karyotype	Normal	10	33.3	5	45.4	1.73	2	0.420
	Complex	15	50.0	3	27.3			
	Other cytogenetic abnormalities	5	16.7	3	27.3			
Risk stratification based on cytogenetic	Favorable	0	0.0	1	9.1	4.36	2	0.113
	Intermediate	14	46.7	7	63.6			
	Adverse	16	53.3	3	27.3			
Molecular Biology	Yes	37	43.9	12	75.0	1.92	1	0.135
	No	29	56.1	4	25.0			
The presence of mutations	Yes	3	8.1	2	16.7	0.72	1	0.356
	No	34	91.9	10	83.3			
Risk stratification based on molecular biology	Favorable	2	5.4	1	8.3	0.45	2	0.798
	Intermediate	34	91.9	11	91.7			
	Adverse	1	2.7	0	0.0			
ECOG	0-1	29	43.9	12	75.0	4.97	1	0.024
	2-4	37	56.1	4	25.0			
LVEF	Normal	35	53.0	13	81.3	4.23	1	0.035
	Abnormal	31	47.0	3	18.7			
CCI	4	3	4.5	1	6.3	0.08	1	0.588
	>4	63	95.5	15	93.8			
HCT-CI	1-2	9	13.7	3	18.7	0.51	2	0.774
	3-4	22	33.3	6	37.5			
	>4	35	53.0	7	43.8			
	≥50%	23	34.8	4	25.0			

Karyotype was not significantly associated with treatment decisions (p = 0.420), but patients with a normal karyotype (45.4%) received a second line more often than those with complex cytogenetics (27.3%). Most patients classified with adverse cytogenetic risk (53.3%) received only a single line of therapy. By contrast, a significant proportion of patients who underwent a second line of treatment (63.6%) were categorized within the intermediate-risk group. Only one patient identified as having favorable risk underwent a second line of therapy. While the distributions among the groups showed variation, these differences did not reach statistical significance (p = 0.420 for karyotype type and p = 0.113 for cytogenetic risk). The results show that age is an important factor influencing the number of therapeutic lines. Nevertheless, karyotype, performance status, and cardiac function still influence clinical decisions.

The death rate for the entire study group was 90% (74/82 patients), of which 21.6% (16/74 patients) died less than 60 days after the start of treatment. Among individuals who died within 60 days, the leading cause of death was infectious diseases, which accounted for 12 of the 16 cases. This was followed by cardiovascular causes, and lastly, hemorrhagic causes. In the case of patients who survived more than 60 days and who underwent only a single line, the death rate was 86% (43/50 patients), and in the case of patients who underwent two lines, the death rate was 94% (15/16 patients).

For single-line treatment, patients treated with Aza and Dac had higher mortality odds, with Aza at 1.26 (p = 0.082) and Dac at 1.24 (p = 0.067), compared to those receiving HMAs+Ven. In the two-line treatment group, patients treated with LDAC had a higher odds ratio for death (1.13), but this was also not statistically significant (p = 0.285).

The analysis of mean and median survival times according to the number of treatment lines revealed a longer survival in patients who received two lines of therapy compared to those treated with only one line, as Table 7 displays.

**Table 7:** Kaplan-Meier analysis: mean and median survival times by the number of treatment lines

Treatment	Mean (estimate)	Std. error	95% CI lower	95% CI upper	Median (estimate)	Std. error	95% CI lower	95% CI upper
One line	10.24	1.28	7.74	12.75	7.00	1.51	4.04	9.96
Two lines	17.59	2.97	11.77	23.41	11.00	4.00	3.16	18.84
Overall	12.30	1.34	9.68	14.92	8.00	0.87	6.29	9.71

The log-rank (Mantel-Cox) test showed a statistically significant difference in median OS based on the number of treatment lines (χ² (df = 1) = 4.30; p = 0.038). Additional tests, including Breslow and Tarone-Ware, confirmed the statistical significance of this difference, as Table 8 displays.

**Table 8:** Overall comparison of survival distributions by the number of treatment lines Abbreviations: chi-square test: χ2—test value, df—degree of freedom, p-value.

Test	Chi-square	df	p-value
Log-rank (Mantel-Cox)	4.30	1	0.038
Breslow (generalized Wilcoxon)	5.59	1	0.018
Tarone-Ware	5.26	1	0.022

Median and mean survival times were slightly higher in the group treated with HMA plus venetoclax compared to those receiving azacitidine or decitabine alone, as shown in Table 9.

**Table 9:** Kaplan-Meier analysis: mean and median survival times by treatment type

Treatment	Mean (Estimate)	Std. Error	95% CI Lower	95% CI Upper	Median (Estimate)	Std. Error	95% CI Lower	95% CI Upper
Aza	9.85	2.27	5.40	14.30	7.00	1.80	3.48	10.52
Dac	9.50	1.83	5.92	13.08	6.00	1.17	3.71	8.29
HMA+V	11.27	1.77	7.80	14.73	9.00	2.05	4.97	13.03
Overall	10.55	1.31	7.97	13.12	7.00	1.04	4.97	9.04

However, statistical tests comparing survival distributions between treatment types did not reveal significant differences, as Table 10 displays.

**Table 10:** Overall comparison of survival distributions by treatment type Abbreviations: chi-square test: χ2—test value, df—degree of freedom, p-value.

Test	Chi-square	df	p-value
Log-rank (Mantel-Cox)	1.58	2	0.455
Breslow (generalized Wilcoxon)	2.21	2	0.332
Tarone-Ware	1.98	2	0.372

**Figure 2:**
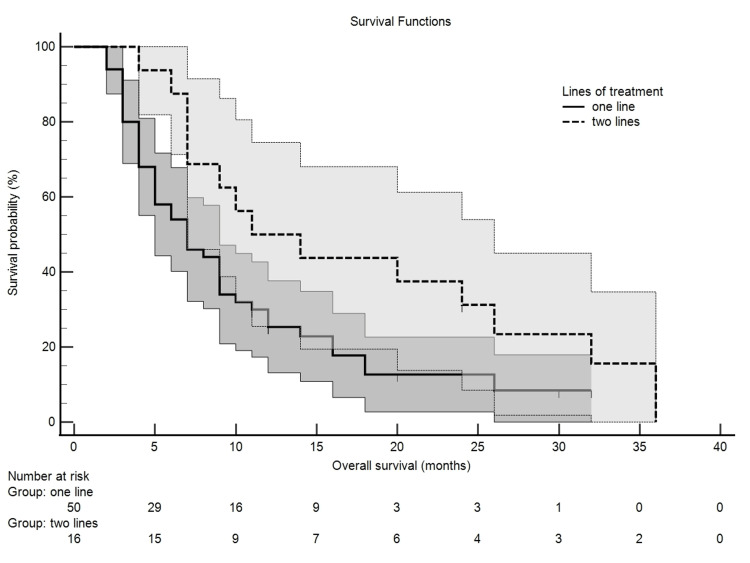
Kaplan-Meier survival curves comparing overall survival (OS) by number of treatment lines. Median OS was 7.0 months for patients receiving one line of therapy and 11.0 months for those receiving two lines, with 95% confidence intervals shown.

According to the Kaplan-Meier survival curves, presented in Figure 2, patients receiving two lines of treatment had a longer median survival (11.00 ± 4) than those treated with a single line (7.00 ± 1).

## Discussion

Based on our study, decitabine was the most commonly used hypomethylating agent. Azacitidine and decitabine demonstrated comparable response rates; however, a greater proportion of patients achieved therapeutic responses with azacitidine (50% vs. 46.9%). Nevertheless, more than 50% of patients in both treatment groups exhibited no response to the therapy, underlining the necessity for the development of additional therapeutic strategies for elderly patients diagnosed with AML.

The prognostic analysis revealed that 97.6% of patients were categorized as having an intermediate-adverse risk profile, with a p-value of 0.001, as complex karyotypes were associated with poorer responses and higher-risk classifications.

In cases where patients did not achieve a response, transitioning to an alternative HMA has been associated with extended survival. We notice significant differences in OS outcomes when comparing our study findings with the existing literature. In our cohort, the median OS after a single treatment was seven months, which increased to 11 months for patients who received a second-line treatment. This variation can be attributed to several factors. In addition, our data suggest that factors such as ECOG performance status, age, and LVEF significantly influence survival outcomes. The improved transfusion independence observed in patients receiving venetoclax supports its added value, particularly in frail patients who may benefit from rapid hematologic improvement. These findings align with recent studies emphasizing the role of venetoclax in improving the quality of life and early responses in elderly AML patients.

 Patients receiving frontline treatment often had high-risk cytogenetics, secondary AML, and considerable comorbidities, which made them more susceptible to early mortality. By contrast, the patients who received second-line treatment had already survived frontline therapy, suggesting a selection bias in favor of those with greater disease resilience or better tolerance to treatment. It should be noted that patients receiving second-line therapy had more favorable clinical profiles, including better ECOG performance status, which may have influenced the improved survival observed in this subgroup. Second-line therapy often involved modifications, such as switching to HMAs, LDAC, or adding venetoclax, which improved response rates and increased transfusion independence. The combination of venetoclax with hypomethylating agents improved survival to 11 months, although the small number of cases in this subgroup limited statistical significance. Furthermore, many frontline patients experienced early disease progression or treatment intolerance, reducing the likelihood of receiving further therapy. These aspects may explain the survival disparity. Moreover, our results highlight that patients with favorable ECOG scores (0-1), normal left ventricular ejection fraction (LVEF), and age under 75 were more likely to achieve a therapeutic response and survive beyond six months. These parameters, already recognized as prognostic factors in AML, were statistically validated in our cohort. Transfusion independence, particularly in Venetoclax-treated patients, further reinforces the association between early hematologic recovery and improved outcomes. These elements may serve as useful markers for treatment stratification in real-world settings.

Data from the literature suggest higher survival rates with hypomethylating agents. The median OS with decitabine was reported as 7.7 months, while azacitidine alone improved OS to 10.4 months - values that exceeded the survival outcomes observed in our study [18,19]. Furthermore, the combination of azacitidine and venetoclax demonstrated a significantly improved OS of 14.7 months, as reported in the VIALE-A trial, a higher result than the 11-month OS recorded in our study. However, real-world studies suggest that clinical outcomes may be worse than those in controlled trials [20,21]. The survival differences between our study and the literature can be attributed to factors such as patient selection, sample size, and cytogenetic and molecular testing availability. While the VIALE-A study applied strict inclusion criteria, our study included a more diverse and high-risk population, which may explain the lower survival rates.

There are a few limitations to the study that deserve consideration.

Given its retrospective and single-center design, caution must be exercised when interpreting the findings, as they reflect a single institution's clinical realities and treatment patterns. The small sample size, especially in the venetoclax subgroup, limited the power to detect statistically significant differences.

In addition, the heterogeneous population, composed predominantly of elderly and frail individuals with multiple comorbidities, created challenges in both treatment intensity and survival interpretation.

Moreover, the lack of access to standardized molecular platforms limited comprehensive genomic profiling, which may have affected the depth of prognostic stratification. Of note, all patients who received LDAC as part of their initial treatment died, and 9 of them died within the first 60 days of treatment. Among the patients who died due to infectious complications, confirmed bacterial sepsis was the most frequently identified cause. Pulmonary infections were also observed in neutropenic patients, although microbiological confirmation was not always available due to the retrospective nature of the study. Cardiovascular-related deaths included acute decompensation in patients with pre-existing heart disease, such as heart failure and ischemic episodes. Nevertheless, the study offers valuable real-world insights into the use of hypomethylating agents and the potential utility of Venetoclax in older patients with AML.

Future strategies to improve clinical outcomes in this population could include broader implementation of early molecular profiling to tailor better treatment approaches, closer monitoring for infection-related complications in neutropenic patients, and including patient-centered parameters such as quality of life and functional independence in treatment selection. Expanding access to newer agents-such as menin inhibitors (targeting epigenetic dysregulation), FLT3 inhibitors (addressing FLT3-mutated AML), and IDH1/2 inhibitors (used in AML with isocitrate dehydrogenase mutations)-and outpatient-based therapy protocols could also improve adherence and tolerance in frail populations.

Despite these limitations, some encouraging findings emerged from the study. A higher proportion of patients who responded to treatment, particularly those receiving venetoclax in combination with azacitidine or decitabine, achieved transfusion independence. This highlights the clinical benefit of effective AML therapy in terms of improving patients’ quality of life [22].

Clinicians treating elderly patients with AML should consider using hypomethylating agents, specifically azacitidine or decitabine, in combination with venetoclax as the preferred first-line treatment. This approach is particularly recommended for individuals with favorable Eastern Cooperative Oncology Group (ECOG) performance scores of 0 to 1 and those who have preserved cardiac function. Due to the high risk of early mortality associated with low-dose cytarabine (LDAC), its use should be limited or approached with caution. These recommendations align with current treatment trends and are supported by the outcomes observed in our patient cohort.

## Conclusions

This study reinforces the role of hypomethylating agents combined with venetoclax as an emerging standard of care for elderly AML patients who are unfit for intensive chemotherapy. Integrating venetoclax into treatment regimens appears promising, enhancing survival outcomes and transfusion independence in responders. The study population consisted exclusively of elderly patients ineligible for intensive therapy, ensuring consistency in the clinical profile and relevance of the findings. These results are consistent with real-world data and underscore the value of early hematologic recovery, especially in frail individuals.

However, many patients failed to respond, underscoring the urgent need to develop more effective therapeutic options. The limited efficacy of LDAC and the modest survival seen with single-agent hypomethylating therapies highlight the significant therapeutic challenges still present in this patient group. Future efforts should optimize risk stratification, expand access to innovative treatments, and adapt therapeutic approaches to clinical performance status and comorbidity burden, which have proved to be critical prognostic factors in our cohort.
